# Consumer-Mediated Data Exchange for Research: Current State of US Law, Technology, and Trust

**DOI:** 10.2196/12348

**Published:** 2019-06-03

**Authors:** Yiscah Bracha, Jacqueline Bagwell, Robert Furberg, Jonathan S Wald

**Affiliations:** 1 Division of eHealth Quality and Analytics RTI International Research Triangle Park, NC United States; 2 InterSystems Cambridge, MA United States

**Keywords:** health records, personal, electronic health records, patient access to records, research, trust, data collection, consumer health information

## Abstract

A compendium of US laws and regulations offers increasingly strong support for the concept that researchers can acquire the electronic health record data that their studies need directly from the study participants using technologies and processes called consumer-mediated data exchange. This data acquisition method is particularly valuable for studies that need complete longitudinal electronic records for all their study participants who individually and collectively receive care from multiple providers in the United States. In such studies, it is logistically infeasible for the researcher to receive necessary data directly from each provider, including providers who may not have the capability, capacity, or interest in supporting research. This paper is a tutorial to inform the researcher who faces these data acquisition challenges about the opportunities offered by consumer-mediated data exchange. It outlines 2 approaches and reviews the current state of provider- and consumer-facing technologies that are necessary to support each approach. For one approach, the technology is developed and estimated to be widely available but could raise trust concerns among research organizations or their institutional review boards because of the current state of US law applicable to consumer-facing technologies. For the other approach, which does not elicit the same trust concerns, the necessary technology is emerging and a pilot is underway. After reading this paper, the researcher who has not been following these developments should have a good understanding of the legal, regulatory, technology, and trust issues surrounding consumer-mediated data exchange for research, with an awareness of what is potentially possible now, what is not possible now, and what could change in the future. The researcher interested in trying consumer-mediated data exchange will also be able to anticipate and respond to an anticipated barrier: the trust concerns that their own organizations could raise.

## Introduction

### How Researchers Now Acquire Electronic Health Records

Researchers who need electronic health record (EHR) data [1] for study participants receiving care in the United States commonly acquire them from health care providers who agree to extract the data from their EHR databases. This data acquisition method is convenient and suitable for studies that need data only from participants who receive care from cooperative providers. However, the method is pragmatically infeasible for studies that require complete longitudinal records from all providers caring for participants who individually and collectively receive care from multiple providers. Examples include the National Institutes of Health All of Us Research Program (formerly the Precision Medicine Initiative) [2] and studies in which participants are patient members of Patient-Powered Research Networks [3]. Researchers could not feasibly establish and maintain data-sharing relationships with all the geographically dispersed providers who could be caring for all participants [4-7], including providers who may not have the capability, capacity, and/or interest in supporting research [8].

Theoretically, researchers could overcome these logistical challenges by obtaining comprehensive longitudinal electronic patient records from a clinical data research network, a clinical data repository, and/or a health information exchange, but practically, both convenience and data completeness challenges remain [9,10]. Currently, these types of organizations assemble records for patients who receive care within a specific geographic region, limiting utility for studies that enroll participants nationally. In the future, the National Trusted Data Exchange Framework (Exchange) may compile records across geographic areas, but this does not help researchers who need data now and will not help researchers who are unable to access the Exchange. Even if completeness and access challenges were resolved, there still is the challenge of linking records for the same patient across multiple providers [11,12].

### Consumer-Mediated Data Exchange as a Data Acquisition Alternative

Consumer-mediated data exchange [13,14] may offer researchers a way to acquire the EHR data they need without confronting these logistical barriers. There are 2 approaches. In one, which we call *Download and Send*, study participants use a consumer-facing app to download and aggregate their own health records, which they then contribute to the research database. In the other, which we call *Transmit*, study participants use an app that directs their providers to transmit their data to the research database.

### Legal Support for Consumer-Mediated Data Exchange

There has been strong and enduring US federal support for the principle that individuals should have access to their own EHRs and that individuals can share their records with third parties, including researchers [15-18]. Legal support was first codified through a 2011 amendment to the Health Insurance Portability and Accountability Act (HIPAA), which requires US providers and health plans to fulfill patient requests for their own data and to provide the data in electronic form if the patient requests [19]. The 21st Century Cures Act states that consumers must be able to access their own electronic health information “with no special effort” [20]. The current administration’s MyHealtheData initiative is predicated on the belief that all individuals should have access to their electronic health information and be empowered to use them however they wish [21,22].

To implement these principles, the Centers for Medicare and Medicaid Services (CMS) had incentivized providers to give patients the ability to view, download, and transmit their own data electronically, originally through the Meaningful Use program [23] and, now, through the Hospital Inpatient Prospective Payment Systems [24] and for every model within the Quality Payment Program, including the Merit-Based Incentive Payment Systems (MIPS), and advanced alternative payment models [25]. As of 2019, CMS requires providers participating in these programs to use health information technology that meets the most recent (2015) certification criteria established by the Office of the National Coordinator for Health Information Technology (ONC). These criteria not only require the technology to allow the patient to view, download, and transmit their own records manually, they also require the use of *open application programming interfaces* (APIs), which allow different technologies to exchange information with each other. APIs offer the technical foundation for patient access to their own electronic health information without special effort.

CMS and ONC took another major step in February 2019 when both agencies released notices of proposed rulemaking, on the same day, intended to accelerate the interoperability of electronic health information in the United States by leveraging consumer-mediated data exchange [26,27]. CMS proposes regulations that would give all publicly insured consumers access to their own claims data. The ONC proposes to change some of the criteria that certify the APIs used in provider-facing technology, where the proposed changes would make it much easier for consumers to access and use their own EHR data with the assistance of any consumer-facing app. Both proposed rules are intended to promote interoperability, and both agencies consider consumer-mediated data exchange to be a linchpin in that effort [28,29]. Although the major objective of interoperability is to improve care and outcomes at a lower cost, federal pronouncements also reference researchers’ enhanced ability to acquire the data they need.

In summary, in the United States, there is strong legal and regulatory support for the principle that consumers have a right to access their own electronic health information and that consumers can use their data however they wish, including contributing them to a research database.

### Potential Barriers Researchers May Encounter When Using Consumer-Mediated Data Exchange

For consumer-mediated data exchange for research to be viable at scale, the following conditions must exist:

#### Technical

All study participants must receive care from providers who have the technical capability to respond to patient requests either to download their own data (approach 1: Download and Send) or to direct their own data to a researcher (approach 2: Transmit). In addition, there must be consumer-facing technologies to support the participant.

#### Utility

The data obtained through this method must be useful for the study.

#### Trust

The participants, providers, researcher’s organization and Institutional Review Board (IRB) must have any potential trust concerns allayed. This paper focuses on trust concerns that the researcher’s organization or IRB could raise, because if they are not addressed, they may refuse to approve the study. (If providers have trust concerns, they will not make this service available to their patients, which for this paper is indistinguishable from a technical barrier. If prospective participants have trust concerns, they would decline to participate, and presumably, there would be others without such concerns for the study to proceed).

### Structure of This Paper

This paper first reviews the contemporary technology landscape to assess the level of technical support for both approaches to consumer-mediated data exchange, along with the types of data that researchers could expect to receive. The paper does not address data completeness, provenance, harmonization, and other utility barriers in using EHR data for research, as these have been documented elsewhere [1,8,30-33].

The paper will explain why technical viability in theory is now strong for the *Download and Send* approach and weak but rapidly growing stronger for the *Transmit* approach. The paper outlines the trust concerns that could arise with *Download and Send* and offers suggestions to researchers for responding to them. The paper then describes regulatory and technology developments currently underway that should make the *Transmit* approach more viable in the future, which would mitigate many trust concerns that currently exist.

The paper concludes with a summary, limitations, and a review of potential future developments.

## Technical Support for Consumer-Mediated Data Exchange

### Prevalence Among Providers of Necessary Provider-Facing Technology

#### Approach 1: Download and Send

For the *Download and Send* approach to be viable, all US providers must be able to give patients the ability to download their records electronically, if the patient requests. This means that, at a minimum, providers have the necessary technology to recognize and respond to a patient-initiated *download* request.

The ONC had estimated that, in 2015, 87% of hospitals and 41% of office-based physicians gave patients the ability to download their own medical records [34,35], representing a sharp and steady increase from previous years. In 2012, only 14% of hospitals reported having this ability [35,36]. The change in prevalence of download capabilities for office-based physicians is less clear as, in 2013, the ONC reported that 33% offered patients all 3 *view, download, and transmit* capabilities [37], without reporting the percentage that only had download capabilities.

Since the 2015 reports, estimates of provider prevalence with capabilities have continued to increase. In 2018, the ONC reported [38] that among MIPS-eligible hospitals and clinicians, more than 90% of hospitals and more than 80% of US clinicians were using health information technology systems, meeting the most recent (2015) certification requirements [39]. These requirements include giving patients the ability to download their own records, where the data types must include those in the Common Clinical Data Set [40,41].

In a 2017 report to the Congress, the US Government Accountability Office reported that most providers achieved the view, download, and transmit functionality through patient portals [42]: The patient logs into the portal with a username and password and from the portal clicks a *download* button. The patient can download a PDF of their data to a drive, or if they are being assisted by a personal health records app (more below), they can download the data to the app which may offer added services such as configuring the data for visualization and manipulation. From a configured portal, the patient could also click a *transmit* button to send records to another provider, the approach to be considered next.

On the basis of ONC estimates, it is likely that most, if not all, US providers either currently (March 2019) have the *download and send* capability or will soon have it. On the basis of the Government Accountability Office report, it is likely that, through 2016, providers offered these capabilities through patient portals. We have discussed the use of APIs as an alternative technology in more detail below.

#### Approach 2: Transmit

For the *Transmit* approach to be viable, all US providers must give patients the ability to electronically direct their providers to transmit their data to a designated recipient.

In 2015, the ONC reported that 66% of hospitals [43] and 19% of physician offices [35] had the technical ability to transmit patient records to other providers; the ONC did not comment upon providers’ ability to transmit records to nonproviders such as researchers. We expect the number of providers with transmit capabilities to increase because of the rules that CMS finalized in the fall of 2018: as of 2019, all providers participating in CMS value-based purchasing programs must use health information technology meeting ONC’s most recent (2015) certification requirements [25]. Such technology not only gives patients the ability to download their records but it also gives them the ability to direct their providers to *transmit* their records to a third party, where the transmission can occur using secure Direct Messaging, or by email if the patient requests [39].

Transmission by insecure email would likely be unacceptable to researchers. Transmission by Direct Messaging, however, requires the electronic address of the recipient, which the patient must know and be able to share with the transmitting provider.

This is problematic for usability. Hospitals’ most commonly reported barrier to electronic information exchange was difficulty locating the electronic addresses of the recipient [44], a finding supported by an ONC user experience study [45]. Ideally, there would be a national directory that consumers and providers could search to locate the recipient address and just click to make it happen, and ideally, researchers would be able to enter their study names and addresses into this directory.

Provider directories containing digital contact information exist in both the private and public domains. The nonprofit organization, DirectTrust, maintains a directory [46], which we will not discuss further because there is no federal agency with authority over its structure or contents.

Publicly, CMS maintains a directory called the National Plan and Provider Enumeration System (NPPES) [47-49], originally established as a mechanism for HIPAA-covered entities to exchange information with each other. CMS assigns a unique numeric National Provider Identifier to every individual clinician and facility submitting claims to CMS, and these numeric identifiers represent the provider entries in the NPPES. Provider entries contain name, taxonomy code and description, mailing address and practice address. In response to requirements in the 21st Century Cures Act, CMS modified the NPPES [47-49] to accept digital contact information called *endpoint identifiers* [50,51]. However, not all providers list their endpoint identifiers in the NPPES. In its February 2019 proposed rule, CMS proposes to address this problem by creating and publicly disseminating a list of providers *without* endpoint identifiers in the NPPES, to exert publish pressure upon them.

With respect to researcher inclusion in the NPPES, CMS directions for obtaining an identifier, and for becoming listed in the NPPES, convey through use of examples such as “physician” and “hospital” that CMS uses the word “provider” to refer to an individual or organization that delivers medical care. However, some individuals and organizations now listed in the NPPES have research-related taxonomy codes. Due to the novelty of consumer-mediated data exchange, we doubt that these providers are receiving consumer-directed electronic health information. Rather, we believe that their presence in the NPPES facilitates administrative mechanisms required to charge a research study when its participants receive clinical care. However, the fact that research-related individuals and organizations already appear in the NPPES demonstrates that researchers interested in receiving consumer-directed electronic health information do have a mechanism for listing themselves in this public directory.

Usability challenges do remain but could be addressed by the ONC. For example, the ONC already publishes a Patient Engagement Playbook [52] for providers. Chapter 2 offers providers a step-by-step guide for setting up a user-friendly patient portal that patients can use to download their health information. The Playbook is now silent about how providers can set up their portals to enable patients to *transmit* their health information, but it could be amended to include this information. Not only would this facilitate consumer-directed interoperability, it also would facilitate consumer-directed data exchange for research.

This review suggests that although provider-facing technology should support transmit functionality, usability is a barrier, particularly for consumer attempts to direct the transmission of data using portal-based technology. It is possible that these usability issues will be addressed, and it also is possible that the introduction and use of new technology will address usability issues in other ways. Between 2015 and 2019, technology evolved through the maturation and testing of standards that were in development at the time that ONC finalized its 2015 certification requirements. The emerging technology rapidly gaining adoption involves the exchange of electronic health information through *FHIR-based APIs*, using the *OAuth 2.0* authentication protocol. *FHIR* means *Fast Healthcare Interoperability Resources*; this is a relatively new standard for exchanging health care information electronically [53-56]. As noted earlier, an *API* is a technology that permits 2 applications to communicate or exchange electronic information between them [57]. *OAuth 2.0* allows consumers to give their apps access to their private health information without exposing their login credentials, and thus, creating an additional layer of security.

When both provider- and consumer-facing technologies use the FHIR APIs, the OAuth 2.0-protected consumer can easily access his or her data from the provider and then transmit the data to a target of the user’s choice. In theory, the target could be another provider, to facilitate interoperability. The target could be the consumer’s device, which is functionally equivalent to the *download* capability. The target could be a research database: the consumer would still need to know its digital endpoint, and presumably, the research team would give that to its study participants. In other words, consumers could use the new technology to direct their providers to transmit their data to a research database without the need for the researchers to list themselves in the NPPES or any other directory.

For this emerging technology to support research, all US providers must have technology that can support FHIR APIs with OAuth 2.0. The rule that ONC proposed in February 2019 [58,59] is intended to close the remaining gaps to achieve ubiquity. On their own, without regulatory requirements, many vendors in the industry have already adopted these standards. The ONC reports in both its proposed rule [58] and in a 2018 blog [60] that as of mid-September 2018, 51% of certified vendors were already using some version of FHIR APIs and OAuth 2.0 together. On the basis of these vendors’ market shares, the ONC concluded that approximately 87% of hospitals and 57% of MIPS-eligible clinicians had technology with these capabilities. If ONC’s proposed certification criteria for provider-facing APIs [61] are finalized, all vendors serving providers would have these capabilities and ubiquity among providers would be possible by 2021 or 2022.

In summary, to support patient care, most providers now have technology that supports *Transmit*, but *usage* of that technology to transmit electronic health information is underdeveloped. Social and organizational processes could change if all providers listed their digital endpoint identifiers in the NPPES, and once those processes emerged, they could be used to transmit data for research as well as for patient care, if researchers also presented their digital endpoint identifiers in the NPPES. However, researcher use of *Transmit* might be easier if their study participants use consumer-facing technology that leverages FHIR APIs, under the assumption that provider-facing technology uses FHIR APIs as well. The ONC’s 2019 proposed rule is intended to ensure that all providers have FHIR API technology by 2021 or 2022, a very promising development.

#### Summary Regarding Prevalence Among Providers of Necessary Technology to Support Consumer-Mediated Data Exchange for Research

If ONC estimates are correct, it is likely that, as of early 2019, most US providers already have technology capable of supporting the *Download and Send* approach to consumer-mediated data exchange for research. In this approach, study participants would download their own data from all their providers and transfer the aggregated records to the research database.

It is likely that, in a few years, all providers will have technology that has been certified to support the *Transmit* approach to consumer-mediated data exchange for research, where the transmission method relies upon FHIR APIs.

### Existence of Necessary Consumer-Facing Technology

#### Approach 1: Download and Send

The *Download and Send* approach requires the existence of a consumer-facing app that can collect and compile data from multiple providers and continue to collect these data automatically, if the consumer gives this direction. Such apps are often called *untethered* or *standalone* personal health records [62-65]. The word “untethered” means that they operate independently from a particular provider and/or EHR vendor (see Textbox 1). These apps are existent: *App stores* return numerous results when entering the words “personal health records” into the search fields. We call attention to some that are notable.

At least two vendors of consumer-facing apps, both relying on portal download technology, promote their research utility. The vendor Carebox boasts that its app can collect medical records automatically, once the user establishes the connection [66], and reports that the Leukemia and Lymphoma Society National Patient Registry [67] is using its technology to enable patients to download and aggregate their records for contribution to the registry. Hugo, a commercial app developed by the vendor Me2Health was specifically designed to enable consumers to download and aggregate their own EHRs and send them to researchers [68,69]. Investigators at the Yale Center for Clinical Investigation and the Mayo Clinic are conducting a pilot to explore Hugo’s utility for postmarket surveillance of medical devices [70]. Both Carebox and Me2Health take responsibility for transferring the aggregated records to the researchers, using whatever transfer protocols the researcher requires. Both apps give users the ability to visualize their own data on the device, to automatically update with new records whenever they appear, and to sever the connection whenever they wish.

Consumer-facing apps that use FHIR APIs conceivably can access the consumers’ electronic health information from providers and then send them to a designated target, which includes the consumer’s own device, which is functionally equivalent to the *download* technology. In January 2017, the vendor PatientLink Enterprises won a 2016 ONC competition [71,72] for its product, MyLinks [73], which uses the FHIR API to aggregate data from multiple health care systems. A year later, in January 2018, Apple unveiled a beta version of a personal health record iPhone app using an FHIR API [74], an event that attracted a great deal of attention among analysts who watch the health information technology industry [75-78]. Data exchanged using Apple’s app do not traverse Apple’s network, a feature that Apple cites prominently on its website [79]. This is a key differentiator compared with portal download technology and is discussed in this paper in more detail below, in the section titled “Trust Concerns that Research Organizations or IRBs Could Raise.”

**Personal health records: coevolution of technology and lexicon.**

When patient portals were first introduced, they were called personal health records. Later, when consumer-facing applications that gathered data from multiple sources entered the market, these too were called personal health records. To differentiate between them, market analysts and some researchers began referring to the patient portal as a tethered personal health record and referring to the consumer-facing application as an untethered personal health record. As patient portals became more common, and included convenience functions such as making appointments online, paying bills, sending secure messages to providers, the vocabulary changed again, and writers simply referred to patient portals as “patient portal”. Once the word “tethered” was less often used to refer to patient portals, its antonym, “untethered”, was less often used to refer to consumer-facing applications that can compile data from multiple sources.

#### Approach 2: Transmit

None of the apps that we reviewed, which rely on portal technology, advertise any ability to help the user direct their providers to transmit their data to a third party, either other providers or a research database. Apps that use FHIR APIs could authorize providers’ technology to transmit the data to other providers or to a research database. There are growing numbers of consumer-facing apps that use FHIR APIs and one has been designed to support data transmission for research. This is the app designed and built for the Sync for Science project, on behalf of the All of Us Research Program. We have discussed Sync for Science in more detail below, in the context of future possibilities.

#### Technology Summary

As shown in Table 1, as of March 2019, there is strong technical support for the *Download and Send* approach and limited but growing support for the *Transmit* approach. The next sections focus on the trust concerns associated with *Download and Send.*

**Table 1:** State of provider- and consumer-facing technology to support consumer-mediated data exchange for research, as of March 2019.

Approach	Provider-facing technology	Consumer-facing technology
Approach 1: Download and send	Likely ubiquitous or nearing ubiquity among US providers, using download capabilities from the patient portal.	Many consumer-facing applications on the market tha give users ability to download records and compile them. Two vendors are known to take responsibility for transmitting users’ records to researchers, with user consent.
Approach 2: Transmit	Capabilities from the patient portal exist but insufficient use of digital contact information poses usability barriers. Many providers have technology that uses FHIR APIs^a^, and this technology would support the approach, but there are social, business and usability obstacles. These could be removed within several years if ONC’s^b^ February 2019 proposed rule is finalized.	There are no indications that applications exist in the consumer-facing market that support “transmit” from the portal technology.
		A growing number of applications, with Apple as a market leader, use the FHIR API technology which could be deployed for “transmit”. Sync-For-Science is a prominent pilot testing the technology for research use, but scale is currently limited to four EHR^c^ vendors and approximately 12 providers.
		This holds great promise for the future, but current opportunities are limited.

^a^FHIR API: Fast Healthcare Interoperability Resources open application programming interface.

^b^ONC: Office of the National Coordinator for Health Information Technology.

^c^EHR: electronic health record.

## Trust Concerns That Research Organizations or Institutional Review Boards Could Raise

### Definition of “Trust”

As a social concept, *trust* conveys perceptions of safety, reliability, risk, and vulnerability across a wide range of contexts. It has been modeled as a 3-part function in which *A* (trustor) trusts *B* (trustee) to fulfil *C* (task) [80]. Within clinical information systems, technical representations of social trust facilitate the exchange of protected health information [81], such as credentialing for access control. Trust is necessary to facilitate participants’ involvement in contemporary clinical research studies, such as the *All of Us Research* Program [82].

For this paper, there are 3 actors (participant, researcher, and vendor of the consumer-facing app), and thus, there are 3 trust relationships:

1. Relationship 1: *The study participant trusts the researcher to keep his or her data safe, and not allow the data to be used in any way other than what the participant intends.*
2. Relationship 2: *The researcher trusts the vendor of the consumer-facing app to not permit the data to be used in any way other than what the researcher intends*.
3. Relationship 3: *The study participant trusts the vendor of the consumer-facing app to not permit the data to be used in any way other than what the participant intends*.

All relationships could be violated if the vendor uses participant data in ways that neither the researcher nor the patient authorized [83].

### Why Research Organizations and Institutional Review Boards May Not Trust Consumer-Mediated Data Exchange

In the most common current use of existing technology, which does not rely on FHIR-based APIs, consumers who use commercial personal health record apps to download their data from providers must give the app necessary credentials for accessing their data, such as a patient portal username and password. In addition, the vendor will be exposed to the data during the process of transferring them from the provider to the patient’s device. The vendor will also be exposed if the vendor takes responsibility for transferring the records from the consumer’s device to the research database. Thus, the developers of the consumer-facing apps have access to personal health information that they have the potential to abuse, raising concerns about the integrity of the trust relationships.

These concerns have been enduring [84]. A 2007 study commissioned by the ONC reviewed privacy policies of 30 vendors, finding that none had policies that named the vendors’ data partners or other secondary data users nor did any of the policies explicitly describe what data elements might be shared [85]. Moreover, 5 years later, in 2012, newly published studies showed that personal health record apps available at the time frequently lacked basic security features, with highly variable privacy policies [86,87].

As there is little disagreement about the basic privacy and security protections to which personal health record apps should adhere [88], in 2018, the ONC disseminated an updated model privacy notice [89,90], encouraging vendors to adhere to it. The astute reader will notice the use of the word “encourage” rather than “require”. This is because the ONC has no authority to *require* commercial vendors of consumer-facing products to comply with anything.

No other US federal agency has this authority either. The Office of Civil Rights and the Federal Trade Commission (FTC) are the 2 federal agencies with the closest authority. The Office of Civil Rights investigates every reported HIPAA violation and has the authority to impose fines or other punishments for violations. However, by definition, commercial vendors of untethered health record apps are not HIPAA-covered entities [91], and so they fall outside the authority of the Office of Civil Rights [92-94].

The FTC has authority to investigate complaints of consumer harm, although unlike the Office of Civil Rights, which investigates every reported HIPAA violation, the FTC chooses which complaints to investigate, typically on the basis of the magnitude of harm [86]. The FTC also has authority to impose punishments. However, the FTC only has authority to investigate if a vendor fails to follow its own privacy policies. It does not have the authority to require the vendor to have a policy nor can it dictate what the policy should be [86].

### Will There Be Changes in US Law Anytime Soon?

Some believe that the FTC and the Office of Civil Rights already have the authority they need [95] and argue that until serious harms have been demonstrated, new legislation might stifle the innovation necessary in a time of rapidly evolving technology [96]. In particular, there has been opposition to expanding HIPAA’s scope to cover personal health record app developers. Spokespersons from the Center for Democracy and Technology and others argue that HIPAA contains built-in limitations because of its original intent and that extensions of HIPAA to cover personal health record apps will be inadequate [91,96-99]. Although there are European [100,101] and other models [84] that US legislators and regulators could theoretically adopt, there have been no bills introduced in the US Congress that follow these leads. The closest example was the 2013 changes to the Omnibus HIPAA bill, which extended HIPAA to business associates of covered entities. However, most untethered personal health record apps do not seek formal business association with covered entities; they direct their attention to consumers instead.

As there are unlikely to be changes in US law that would address these concerns, there now are activities underway to better leverage the FTC’s existing authority. The CARIN Alliance is a prominent multisector group within the health care industry that works collaboratively with US government leaders to promote consumers’ ability to access their electronic health information via open APIs [102]. In November 2018, the CARIN Alliance published a *trust framework and voluntary code of conduct* directed toward developers and vendors of consumer-facing apps [103]. Under the trust framework, when app developers place their products on app stores, they would attest that they adhere to the CARIN Code (Code). The Code is based on the internationally recognized standards and practices for sharing consumer information, including the Code of Fair Information Practices. Developers who publicly attest to adhering to the Code, and then violate it, expose themselves to FTC accountability [104].

The press release announcing the framework and Code says:

For the first time, health care organizations and other organizations can have an enforceable code of conduct for third-party applications not covered by HIPAA to self-attest to in order to access health care data on behalf of consumers.

The CARIN Code has stakeholder support from consumers and caregivers, health information networks, former regulators, app vendors, health care providers, medical home networks, and health plans [105]. The CARIN Alliance is actively working with developers to encourage them to adopt the Code as a part of their process of registering their apps and rolling them out to consumers.

### Will the Office of the National Coordinator for Health Information Technology’s Trusted Exchange Framework and Common Agreement (Exchange) Resolve Concerns?

We believe this is unlikely. The Exchange is intended to be the implementation of a provision of the 21st Century Cures Act which directs the ONC to create a framework and agreement for the exchange of electronic health information between health information networks. As noted above, in January 2018 the ONC released a draft of the Trusted Exchange Framework and Common Agreement [106]. This draft affirmed that consumers seeking their own data could access the Exchange with a commercial app as long as the app complied with Framework provisions. ONC reaffirmed these individual access provisions in its second draft, which it released in April 2019 [107], by creating a distinct category called “Individual Access Services”, defined as services which enable individuals to access and obtain copies of their own electronic health information. In the second draft, ONC says that entities that wish to offer Individual Access Services and thus participate in the Exchange must agree to provisions that are aligned with HIPAA, even if they are not HIPAA-covered entities. This includes a requirement that the entity must publish its privacy practices, with a notice that reflects ONC’s Model Privacy Notice [89].

However, these requirements that ONC would impose upon non-covered entities apply only to entities that wish to participate in the Exchange by offering Individual Access Services. ONC still does not impose any requirements upon non-covered entities that choose not to participate in the Exchange, including noncovered apps that help consumers access their data directly from their providers, rather than health information networks.

### Potential Impact of Trust Erosion on Research Organizations and Institutional Review Boards

The Office of Civil Rights, in early 2016, issued strongly worded Guidance directed toward providers that (1) reaffirmed that providers are legally required to provide patients access to their own EHRs upon request and (2) stated that providers were not liable if, upon complying with a patient request, the patient subsequently placed the privacy and/or security of their own records at risk [108].

Research organizations undoubtedly would welcome comparable guidance that specifies liability if studies *receive* potentially exposed participants’ private health information. However, at the moment, there is no such guidance directed toward research organizations or their IRBs nor is there guidance regarding organizations’ culpability if a researcher uses an unregulated commercial app to gather data from study participants.

This silence can provoke uncertainty for research organizations’ legal and risk departments, and they may contemplate *what-if* scenarios: What if the app’s vendor, a small start-up with few resources, has inadequate security and hackers gain access to participant data? What if the vendor sells consumers’ access credentials and/or their data to a third party? A participant who learns of these violations could seek redress from the research organization, attempting to bring a civil suit for monetary damages or bringing a suit in the court of public opinion, placing the organization’s reputation at risk. In addition, IRBs reviewing the study may question whether participants fully understand the risks associated with using unregulated commercial apps to manage and transfer their personal health information [87,109]. They may refuse to approve studies that use consumer-mediated data exchange because they believe that participants are incapable of granting truly informed consent.

The most likely source of guidance would come from the organization, *Public Responsibility in Medicine and Research* [110]. Its goals are to create a strong and vibrant community of ethics-minded research administration and oversight personnel and to provide educational and professional development opportunities to raise the bar of research administration beyond basic regulatory compliance. It also has formalized professional standards and is active in public policy, offering expert opinion to rule-making and advisory bodies governing the research enterprise. This organization may not now be aware of the potential offered to researchers by consumer-mediated data exchange, as well as its attendant trust concerns, as a January 2019 search in its Knowledge Center on the term “personal health records” returned no results.

In conclusion, research organizations or IRBs could be concerned about studies that acquire EHR data using unregulated vendors. The professional society, *Public Responsibility in Medicine and Research,* is likely to hear of these concerns when more researchers take interest in the promise offered by consumer-mediated data exchange. As it develops best practice guidance for research ethics given these new technology developments, the organization will be able to rely upon the CARIN Alliance trust framework, and its associated Code of Conduct, particularly as the framework and Code gains traction with app developers.

## Researcher Options

### Responses to Trust Concerns About the Download and Send Approach

The trust concerns associated with the *Download and Send* approach are focused on the integrity of the entity that manages the consumer-facing app that participants use to download their data from all their providers and then send the compiled record to the research database.

#### Option 1: Build Trust With a Commercial Developer

The research team could proactively build trusted relationships with commercial developers through the following mechanisms:

- Only consider vendors that have adopted the ONC’s Model Privacy Notice [89] or the CARIN Voluntary Code of Conduct [103]. Developers who pledge to abide by the CARIN Code risk being held accountable by the FTC if they violate that pledge.
- Collaborate with the CARIN Alliance to implement the third phase of its Trust Framework, in which third parties certify apps and their vendors for their adherence to the Code and can also create other certification criteria. Research organizations, for example, could develop criteria related to the vendor’s ability to support research needs, including—if applicable—its willingness and ability to securely transfer user data to the research team if the user consents, and to configure these data so that they are analytically digestible.
- Impose the organization’s security and privacy requirements upon the vendor contractually, detailing the consequences to the vendor if the organization learns that the vendor has compromised participant data.
- Establish monitoring systems for the vendor’s privacy policies and the movement of data through the vendor’s system, intervening if there is suspicious activity.

#### Option 2: Build Your Own Personal Health Record App

A research organization could create its own app that its study participants could use, which would take the vendor out of the mix of trusted relationships, so that trust would only be established and maintained between the study participant and the research team. This increases the burden on the research team, which now has to build and maintain a consumer app. The burden is lower if the study is retrospective, only requiring participant records that exist at the time the study begins. The research team’s burden increases if the study is prospective, meaning that new records must be obtained as they become available, which then means that the researcher will have to maintain the app over time and potentially release upgrades that are compatible with changes in the broader environment. The burden is particularly high if the researcher would like to use the app as an incentive to attract participants, offering them the personal data management capabilities that commercial vendors offer.

If the research team is not troubled by these potential burdens, then building and managing its own consumer-facing app could potentially mitigate trust concerns and allow the study to proceed. A faculty member at the Yale Center for Clinical Investigation did just that, which is how the Hugo app was developed [111].

### Lower Expectations About the Comprehensiveness of the Data to Be Received

If neither of the options mentioned above are viable, the researcher could use an app that relies on the FHIR API technology so that the data can be transmitted from the provider to the user’s device without traversing the vendor’s network. Among the consumer-facing apps that say they use FHIR APIs, Apple is the most well-known, and the ONC cited Apple by name in its 2019 proposed rule. However, using Apple could impose limitations on the study. Apple’s app will only work with an iPhone, which means that the study can enroll only iPhone users. In addition, Apple’s app only downloads data from providers who are members of Apple’s partnership. The partnership has grown steadily since Apple first launched the app in January 2018 [112], and as of April 2019, there were more than 370 partnering providers [113]. The partners are diverse, including individual physicians, small specialty practices, and large systems such as Kaiser in Northern California. This is impressive growth, but the partnership still does not include all providers in the United States. So even if all study participants had iPhones, it is possible and probably likely that for each study participant, there would be incomplete data.

### Watchful Waiting Until the Environment Becomes More Favorable

Researchers unable to work within the existing limitations could prepare themselves for a future that may hold one or more of the following:

#### Apple and Equivalents Enroll All US Providers in “the Partnership”

If Apple’s partnership eventually includes all US providers, then limitations in data only coming from participating providers would be removed, leaving only the limitation of iPhone use. This limitation will also evaporate as other vendors develop and promote consumer personal health record products using FHIR APIs. In the future, all consumers may be able to download their electronic health data to their devices using FHIR APIs, and researchers could adapt to whatever device and app and prospective study the participant prefers.

This still would leave open the challenge of getting the data from the participants’ devices to the research database. It is technically feasible for a consumer-facing research app to use the FHIR API standard to request and receive data from a consumer-facing personal health record app, and although we are unaware of apps that support this now, an enterprising developer could create one in the future.

#### The CARIN Alliance Trusted Framework Becomes Normative

If the CARIN Alliance succeeds in fostering its Trust Framework, and vendors of personal health records self-attest to adhering to its Code of Conduct, and symbols of this attestation appear on app stores and product labels, then the symbol could serve as a *trust flag* that helps consumers and others such as researchers differentiate between products on the basis of their adherence to internationally accepted norms regarding the sharing of consumer information.

#### The Federal Trade Commission Asserts an Intent to Investigate if Vendors Violate a Pledge to Adhere to the Code

The norms around vendor behavior would be strengthened if it were publicly known that the FTC will investigate vendors that violate pledges to adhere to the Code. Research organizations could then be assured that there would be externally imposed consequences upon vendors who fail to uphold the standards of conduct.

### Emergent Technology Tuned for Research

In the *Transmit* approach to consumer-mediated data exchange, health care providers’ technology send the study participant’s patient data directly to a research database upon receiving an order to do so from the participant. This approach reduces, if not eliminates, trust concerns because there is no consumer-facing app that is exposed to the participants’ private health information. If the approach relies on the Direct Messaging protocol from the patient portal, technical support may exist but usability is now weak. If the approach relies on the emerging technology of FHIR APIs coupled with OAuth 2.0 authentication, usability may soar, making the *Transmit* approach much more viable.

#### The Sync for Science Pilot

In the spring of 2016, the National Institutes of Health, in collaboration with the ONC, contracted with Harvard Medical School to create a standards-based open source technology framework called Sync for Science [114,115]. The vision was that EHR vendors could use the framework to augment their patient portals to be able to respond to consumer-facing research apps that ask the provider to transfer the patient’s data to a designated research study. Apps that comply with Sync for Science requirements would not need patients’ portal login credentials nor would they handle personal health information. Instead, the study participant would enter a provider-specific code into the app, which would then route the participant to the provider’s compliant patient portal, which would be able to recognize and act upon data transfer requests.

After several years of design, development, and consultation with IRBs, in September 2018, a pilot began to test the Sync for Science transmit technology on behalf of the All of Us Research Program [2]. The pilot involves 4 EHR vendors and approximately a dozen providers. Participating providers are recruiting up to 100 of their patients first to enroll into All of Us and then to consent to use a consumer-facing research app built by the Sync for Science team. The study participant uses the app to identify his or her provider, and then the app asks the provider’s EHR system to transfer the participant’s EHR data to the study. The provider’s EHR system, which the piloting vendor has modified for Sync for Science, uses a 2-step authentication process, first to validate that the request is coming from a registered app and then to validate that a legitimate patient issued the request. If the authentication process succeeds, the EHR system issues an *access token* to the app that enables it to transfer the data.

At no point does the study participant give the research app his or her portal access credentials nor does the app ever see or manage the participant’s private health information. The app merely facilitates, on behalf of the patient/consumer/participant, the exchange of data between the HIPAA-covered entity and research team. Thus, the approach eliminates the primary sources of trust concerns that the researcher’s organization or IRB may have.

#### Scaling Sync for Science

The pilot described above is intended to test the technology with a limited number of EHR vendors and providers. It will only enroll patients who receive care from one of the pilot’s providers. If that participant happens to receive care from other providers, not involved in the pilot, the EHR data from the other providers will not be available. It is a technical proof of concept, rather than a test of how the *Transmit* approach could work for any study, recruiting any participant and receiving care from any provider(s) in the United States.

For this process to work at scale for any study, all US providers must be served by health information technology that complies with the Sync for Science technical framework. The research team must use a consumer-facing app that has the same technical capabilities as the app built by the Sync for Science team, and the apps’ developers must have registered their products with all Sync for Science-compliant health information technology vendors in the United States. The vendors must extend the product’s registration to all the providers that the technology vendor supports [115]. These systemic requirements may seem onerous. The next section describes how the ONC’s 2019 proposed rule could remove apparent barriers.

#### US Federal Regulations Intended to Stimulate and Support Scale

The Sync for Science technical framework is none other than the FHIR API coupled with OAuth 2.0 authentication. If the ONC’s February 2019 proposed rule is finalized, all vendors of provider-facing health information technology that meet ONC certification requirements will support this framework. CMS will likely require all providers participating in its value-based purchasing programs to use health information technology that satisfies the ONC’s now-proposed certification requirements, and this is the mechanism through which all US providers would acquire the necessary technology. The ONC’s 2019 proposed rule, if finalized, also would remove known technical and business barriers to widespread use of the technical framework. The ONC proposes to require vendors of provider-facing technology to (1) respond within 5 business days to consumer-facing apps’ registration requests, (2) publish technical documentation that enables consumer-facing apps to build connections to the vendors’ APIs, and (3) refrain from charging fees to apps or providers other than the fees designed to differentiate themselves in the market with value-added services. With these and other proposed certification criteria, the ONC intends to break down barriers that now inhibit this technically sophisticated approach to consumer-mediated data exchange.

#### When Will the Future Arrive?

The ONC proposes to require vendors to meet the updated certification requirements no later than 24 months after the rule is finalized. If finalization occurs in mid-2019 with these proposed requirements intact, by the middle of 2021, the provider-facing technology should be available to support the *Transmit* approach to consumer-mediated data exchange, for which the Sync for Science is the most visible example.

With regard to the existence of consumer-facing apps that support the *Transmit* approach for research, the prospects are also promising. Apple and other developers of consumer-facing products already are using the FHIR API standards, currently transmitting EHR data from the provider to the user’s device. It should be feasible to use the same technology to transmit EHR data from the provider to a research database: Only the target has changed.

#### About That Directory...

Previously, we discussed directories containing providers’ digital contact information in the context of the portal-supported *Transmit* approach to consumer-mediated data exchange for research. This approach would require the researchers or studies to list their digital endpoint information in the NPPES, so that providers could transmit data to researchers the same way that they would transmit data to other providers, to support patient care.

Although it is administratively and technically possible for researchers to list themselves in the NPPES, it may not be necessary if the study participant uses a research app with a FHIR API with OAuth 2.0 as its core technology. With this technology, the research app already will tell the provider’s technology where to send the requested data, and so neither the provider nor user will have to look up an address in a directory.

However, while the FHIR API technology may eliminate the need for researchers or their studies to be listed in the NPPES, a directory of providers will still be necessary because the study participants will need to tell the research app who their providers are. In the Sync for Science pilot now underway, a small directory exists that only includes the providers participating in the pilot. In the future, at scale, there will need to be a directory that lists every US provider with the technology to respond to a request to transfer data using an FHIR API.

The NPPES may not be a good vehicle for meeting these research needs. Although it is publicly accessible, it was designed to facilitate exchanges of information between HIPAA-covered entities; it was not designed to meet consumer needs. It contains records for health plans and for individual clinicians and facilities. For each entry, there could be several types of electronic addresses used [47,51]. The data transfer technology will most likely exist at an organizational level, embracing hundreds or even thousands of clinicians and perhaps scores of facilities. It is not clear how a study participant would know how to select the appropriate *provider* for the purposes of transferring data.

However, there is an alternative, in which the research app that study participants will use contain its own provider directory, with a user interface designed to help participants search for their providers and select them when they find them. The apps’ developers could use the content of the NPPES (which is publicly available via download) to populate the consumer-friendly research app directory.

In summary, the FHIR API technology eliminates the need for a directory containing digital contact information for the research database, because the target that receives the data will be identified within the research app that the study participants will use. The FHIR API technology does not eliminate the need for a directory containing a list of US providers, but the task of designing and populating a directory that consumers can use will probably be assumed by the developers of the research apps.

## Conclusions

There are 2 approaches to consumer-mediated data exchange for research. In one approach, which we call *Download and Send*, study participants use a consumer-facing app to download and aggregate their EHR data from all their providers and then send the aggregated record to the research database. In the other approach, which we call *Transmit*, study participants use a consumer-facing app to direct their providers to transmit their data to a research database, where the researcher then aggregates records coming from multiple providers.

As of early 2019, technical support for the *Download and Send* approach is presumed to be strong and usability has been demonstrated. If researchers wish to proceed now using the *Download and Send* approach supported by portal technology, they may encounter trust concerns among their organizations or IRBs, focused on the role of the unregulated consumer-facing app. This paper offers ways in which researchers can respond to concerns that their own organizations may raise, but if the organization is very risk-averse, even these responses may not satisfy them. If this occurs, the researcher may need to relax the study’s requirements for complete data from all possible participants.

Alternatively, the researcher could wait until the environment becomes more favorable. Activities are underway now that could produce a much more favorable environment within several years. These include CARIN Alliance’s efforts to establish a universally accepted trust framework, in which the FTC could investigate actors who publicly commit to adhering to it and then violate that pledge. In addition, the ONC has proposed rules which, if finalized, would stimulate universal adoption among providers of next-wave technologies that support the *Transmit* approach, which mitigates many of the current trust concerns.

For the researcher interested in using consumer-mediated data exchange, there are potential limitations other than those that we already addressed:

- The paper assumes that ONC estimates about the widespread prevalence of the necessary provider-facing technology are correct. As the ONC estimates are based on provider self-reporting and vendor attestation, rather than actual patient experience, this assumption could be incorrect. In addition, the ONC estimates rely on the presence of the necessary technology, not whether the provider is actually using it, or that the patients can use it as well [116].
- The paper assumes that study participants are willing and able to use the necessary consumer-facing technology and that they have credentials that allow them to access their own data from their providers. Although there is evidence that consumers are increasingly taking advantage of patient portals [117], these assumptions may be incorrect.
- The paper assumes that data obtained through consumer-mediated data exchange will contain the data elements in the Common Clinical Data Set [40] because that is what the current regulations require. It is possible that researchers need data elements not in the common set, which would limit their utility to the study. In the ONC’s February 2019 proposed rule, the required data would expand to include clinical notes and provenance, and the rule also establishes a predictable process through which further expansions would occur, so these limitations would likely relax over time. What is more troubling is the possibility that the data which providers actually make available are something less than the regulations require [118].

In the absence of the limitations described above, researchers could be using the *Download and Send* approach now to obtain EHR data for their study participants, assuming they are able to manage the trust concerns that their own organizations or IRBs could raise. It is quite likely that within a few years, researchers could be using the *Transmit* approach, which should mitigate these concerns. Hopefully, this paper gives researchers ways to respond to trust concerns if they arise now and prepare themselves for a future in which the concerns are eased because of the anticipated widespread adoption of emerging technologies.

## Abbreviations

API: application programming interface
CMS: Centers for Medicare and Medicaid Services
EHR: electronic health record
FHIR: Fast Health Care Interoperability Resources
FTC: Federal Trade Commission
HIPAA: Health Insurance Portability and Accountability Act
IRB: Institutional Review Board
MIPS: Merit-Based Incentive Payment System
NPPES: National Plan and Provider Enumeration System
ONC: Office of the National Coordinator for Health Information Technology
